# Clinical characteristics and mortality risk of sarcopenia in interstitial lung disease: a systematic review and meta-analysis

**DOI:** 10.3389/fmed.2026.1955396

**Published:** 2026-10-05

**Authors:** Lijuan Zhou, Xuan Wang, Weiwei Yuan, Yahui Yang, Ting Lu, Lu Guo, Xing He

**Affiliations:** 1 Department of Pulmonary and Critical Care Medicine, Sichuan Provincial People’s Hospital, School of Medicine, University of Electronic Science and Technology of China, Chengdu, China; 2 Department of Nursing, Sichuan Provincial People’s Hospital, University of Electronic Science and Technology of China, Chengdu, China; 3 School of Medicine, University of Electronic Science and Technology of China, Chengdu, China; 4 Department of Pulmonary and Critical Care Medicine, West China Hospital, Sichuan University, Chengdu, China; 5 State Key Laboratory of Respiratory Health and Multimorbidity, West China Hospital, Sichuan University, Chengdu, China

**Keywords:** associated factor, interstitial lung disease, meta-analysis, mortality, sarcopenia

## Abstract

**Objective:**

Sarcopenia, prevalent in interstitial lung disease (ILD), increases the risk of poor outcomes. This study aimed to define the clinical characteristics of ILD patients with sarcopenia (ILD-S) and quantify sarcopenia's impact on mortality.

**Methods:**

We systematically searched PubMed, Embase, Cochrane Library, Web of Science, and Scopus for English studies up to 1 September 2026 reporting clinical variables (mean ± SD) in ILD vs. ILD-S groups and adjusted hazard ratios with 95% confidence intervals (CI) for mortality risk. Subgroup analyses were stratified by sarcopenia diagnostic criteria, ILD subtype, and survival time. Heterogeneity was assessed using Cochran's Q and I^2^ statistics. Sensitivity analyses (leave-one-out) and publication bias assessments (Egger's test, trim-and-fill method) were performed.

**Results:**

Fourteen studies comprising 1,546 participants (628 with ILD-S) were included. Compared with ILD patients without sarcopenia, those with ILD-S were significantly older (WMD = 3.03, 95% CI: 2.12–3.93), had higher Gender-Age-Physiology (GAP) scores (WMD = 0.43, 95% CI: 0.15 to 0.71), and a higher FEV₁/FVC ratio (WMD = 1.97, 95% CI: 0.21 to 3.73, *P* = 0.03). Conversely, the ILD-S group showed lower values for FEV₁% predicted (WMD = −6.95, 95% CI: −9.80 to −4.09), FVC% predicted (WMD = −9.46, 95% CI: −12.04 to −6.88), DLCO% predicted (WMD = −5.87, 95% CI: −11.68 to −0.06), body mass index (WMD = −1.35, 95% CI: −1.89 to −0.82), handgrip strength (WMD = −12.80, 95% CI: −17.02 to −8.58), 6-minute walk distance (WMD = −31.01, 95% CI: −60.05 to −1.97), and appendicular skeletal muscle index (WMD = −1.45, 95% CI: −2.15 to −0.76). No significant difference was observed in smoking history. Sarcopenia was strongly associated with increased mortality risk (HR = 2.02, 95% CI: 1.67 to 2.45), and this association remained stable across different follow-up periods. Sensitivity and publication bias analyses confirmed the robustness of the findings.

**Conclusion:**

Patients with ILD-S exhibit a distinctive profile characterized by older age, higher GAP scores, poorer pulmonary function, lower BMI, reduced muscle strength and mass, and significantly elevated mortality. Systematic recognition of these clinical features may facilitate early identification of ILD-S and inform the development of targeted interventions to improve patient outcomes.

**Systematic Review Registration:**

https://inplasy.com/, INPLASY202570030.

## Introduction

Interstitial lung disease (ILD) comprises over 200 disorders characterized by inflammation and fibrosis of the pulmonary interstitium. ILD primarily manifests as reduced lung volumes, impaired gas exchange, and dyspnea, progressing to respiratory failure and death, posing a significant threat to health. Its prevalence ranges from 6.3 to 71 per 100,000 individuals (1). A European study revealed that mortality rates among ILD patients were 2.04 and 1.02 per 100,000 person-years for males and females, respectively (2). And mortality among ILD patients admitted to the ICU reaches 48% (3), highlighting the imperative to improve prognosis as a pivotal challenge throughout comprehensive disease management. Recent studies identify weight loss and malnutrition as risk factors for poor prognosis in ILD patients (4, 5). ILD patients frequently develop lower limb muscle atrophy and weakness during disease progression (6). Further evidence establishes sarcopenia as an independent predictor of mortality in this population (7). However, comprehensive syntheses elucidating the clinical characteristics of ILD with comorbid sarcopenia remain lacking.

Sarcopenia is a prevalent comorbidity in ILD, with an estimated prevalence of 32.1% (8). Patients with ILD and comorbid sarcopenia exhibit diminished quality of life, reduced 6-minute walk distance (6MWD), and elevated anxiety-depression scores (9), potentially contributing to poor prognoses (10). However, Sridhar M et al. reported that sarcopenia demonstrated a non-significant trend toward increased mortality in ILD patients (11). This persistent controversy challenges sarcopenia's prognostic value in adverse outcome prediction.

Therefore, integrated analysis of clinical features and prognostic impacts in ILD-S patients enhances clinical recognition and disease management, enabling targeted interventions to improve outcomes.

However, consolidated evidence regarding sarcopenia's clinical characteristics in ILD and its impact on ILD outcomes remains limited. We conducted this meta-analysis to elucidate the clinical characteristics and prognostic significance of sarcopenia in ILD patients.

## Materials and methods

This study followed the Preferred Reporting Items for Systematic Reviews and Meta-Analyses (PRISMA) guidelines (12) and was registered on the INPLASY platform (http://inplasy.com; Registration No. INPLASY 202570030).

### Literature search

PubMed, Embase, Cochrane Library, Web of Science, Scopus, and Ovid databases were searched from inception to 1 September 2026. Key search terms included: “Lung Diseases, Interstitial,” “Pneumonitides, Interstitial,” “ILD,” “Idiopathic Pulmonary Fibrosis,” “IPF,” “Sarcopenia,” “Sarcopenic” (detailed search strategies in Supplementary Table S1).

### Eligibility criteria

Inclusion criteria: (1) Age ≥18 years; (2) Diagnosis of ILD based on current guidelines (13–15), incorporating medical history, clinical data, and multidisciplinary discussion; (3) Sarcopenia diagnosis incorporates criteria from the Asian Working Group for Sarcopenia 2019 (AWGS 2019) and the European Working Group on Sarcopenia in Older People 2 (EWGSOP2) and CT imaging-based quantification of skeletal muscle (16–18); (4) Prospective or retrospective cohort studies; (5) Reporting adjusted HR with 95% CI for mortality risk in ILD-S using Cox proportional hazards models; (6) Quantitative data regarding sarcopenia mean ± standard deviation (M ± SD) should be obtainable or convertible through algorithm (19, 20).

Exclusion criteria: (1) Duplicate publications; (2) Case reports, conference abstracts, reviews, meta-analyses, animal/cell studies, commentaries, or letters; (3) Studies unrelated to ILD-S; (4) Insufficient data for effect size extraction; (5) Non-English publications.

### Study groups and outcome assessment

For the purposes of this study, we divided ILD patients into two groups: without sarcopenia (ILD) and with sarcopenia (ILD-S). Clinical variables, expressed as M ± SD, were compared between the two groups. To ensure the robustness and reliability of the pooled estimates, we included a variable in the meta-analysis only when data were available from at least three eligible studies. The primary outcomes were the clinical characteristics of the two groups and mortality in the ILD-S group. Extracted data included first author, publication year, country, study design, ILD subtype, total number of ILD patients, follow-up duration, age, smoking history, sex, gender-age-physiology score (GAP score), forced expiratory volume in one second as a percentage of the predicted value (FEV1% predicted), forced vital capacity as a percentage of the predicted value (FVC% predicted), diffusing capacity of the lungs for carbon monoxide predicted value (DLCO%), forced expiratory volume in the first second/forced vital capacity ratio (FEV1/FVC %), body mass index (BMI), handgrip strength (HGS), 6MWD, skeletal muscle index (ASMI), sample size or effect size, M ± SD, HR, and 95% CI for HR, respectively.

### Quality assessment and data extraction

Two investigators (XW and TL) independently screened studies and assessed the quality of the final included literature using the Newcastle-Ottawa Scale (NOS) (21). The NOS evaluates selection (4 stars), comparability (2 stars), and outcome/exposure (3 stars) domains, with a maximum score of 9 stars. Studies were categorized as high (7–9 stars), moderate (4–6 stars), or low quality (0–3 stars). Relevant data were extracted by WY and YY, including: first author, publication year, country, study design, ILD type, M ± SD for continuous variables, and HR with 95% CI for mortality. Discrepancies were resolved by a third reviewer (HX).

### Data synthesis

Weighted mean differences (WMD) with 95% CI were calculated for continuous clinical characteristics M ± SD. For mortality risk, pooled hazard ratios and their 95% CI were calculated using Cox proportional hazards models. The HR was transformed to the logarithmic scale (log-HR), and the corresponding standard errors were derived from the 95% CI. The transformed effect estimates were pooled using inverse-variance weighting on the log scale. The final estimates were then back-transformed and reported with 95% CI. Heterogeneity was assessed using Cochran's Q statistic and the I^2^ index. A random-effects model (DerSimonian-Laird method) was used if I^2^ ≥ 50% or *p* < 0.05; otherwise, a fixed-effects model (IV method) was applied. Subgroup analyses were stratified by sarcopenia diagnostic criteria(Subgroup 1: EWGSOP2, Subgroup 2: AWGS 2019, Subgroup 3: CT imaging-based quantification of skeletal muscle), ILD subtype (Subgroup 4: ILD patients; Subgroup 5: IPF patients) and adverse outcome follow-up duration (≤1 year; >1 year). Sensitivity analysis was performed by sequentially excluding individual studies. Publication bias was assessed using Egger's test (*p* < 0.05 indicating significance); if present, the trim-and-fill method was used for adjustment. Statistical analyses were performed using Stata 16.0, with *p* < 0.05 considered statistically significant.

## Results

### Study selection and characteristics

The initial search yielded 1,921 records: 80 from PubMed, 159 from Embase, 1,312 from Web of Science, 285 from Scopus, 69 from Ovid, and 16 from Cochrane Library. After removing 482 duplicates, 1,439 records underwent title and abstract screening. We excluded 108 case reports and letters or comments, 60 conference abstracts, 149 reviews or meta-analyses, and 172 animal or cell studies. The full texts of the remaining 950 articles were then assessed. Of these, 819 were excluded because they were not relevant to ILD-S, 10 were not in English, 92 had outcomes not aligned with our study objectives, 14 lacked extractable data, and 1 was a duplicate publication based on the same cohort with different sarcopenia measurements from an already included study. Ultimately, 14 studies were included, with the addition of three studies (7, 8, 11, 22–32). These 14 studies enrolled 1,546 patients, of whom 628 had ILD-S. The PRISMA flow diagram is presented in Figure 1. The study populations were distributed across Japan (*n* = 7), Italy (*n* = 1), the United States (*n* = 1), Turkey (*n* = 1), South Korea (*n* = 1), Spain (*n* = 2), and Mexico (*n* = 1). Ten studies provided comparisons of clinical features between the ILD and ILD-S groups. Specifically, age was reported in 10 studies (8, 22–27, 29–31). BMI was available in 9 studies (8, 22–26, 29–31). Smoking history was reported in 5 studies (22, 23, 25, 27, 31). FEV1% predicted was available in 6 studies (8, 22, 23, 25, 27, 31). FVC% predicted was reported in 8 studies (8, 22–25, 27, 30, 31). FEV1/FVC% was available in 4 studies (8, 22, 25, 31). DLCO% predicted was reported in 8 studies (8, 22–24, 26, 27, 30, 31). GAP score was available in 4 studies (8, 22, 29, 31). HGS was reported in 3 studies (22, 26, 29). The 6MWD was available in 3 studies (8, 26, 30). ASMI was reported in 3 studies (22, 26, 29). Mortality data were extracted from 8 studies (7, 11, 23, 25, 27–29, 32). The NOS assessment indicated that all 14 studies were of high quality, with scores above 7 stars. The main factors affecting the quality scores were insufficient comparability between groups and unreported loss to follow-up or non-response rates (see Supplementary Table S2). Detailed information for all included studies is provided in Supplementary Table S3.

**Figure 1:**
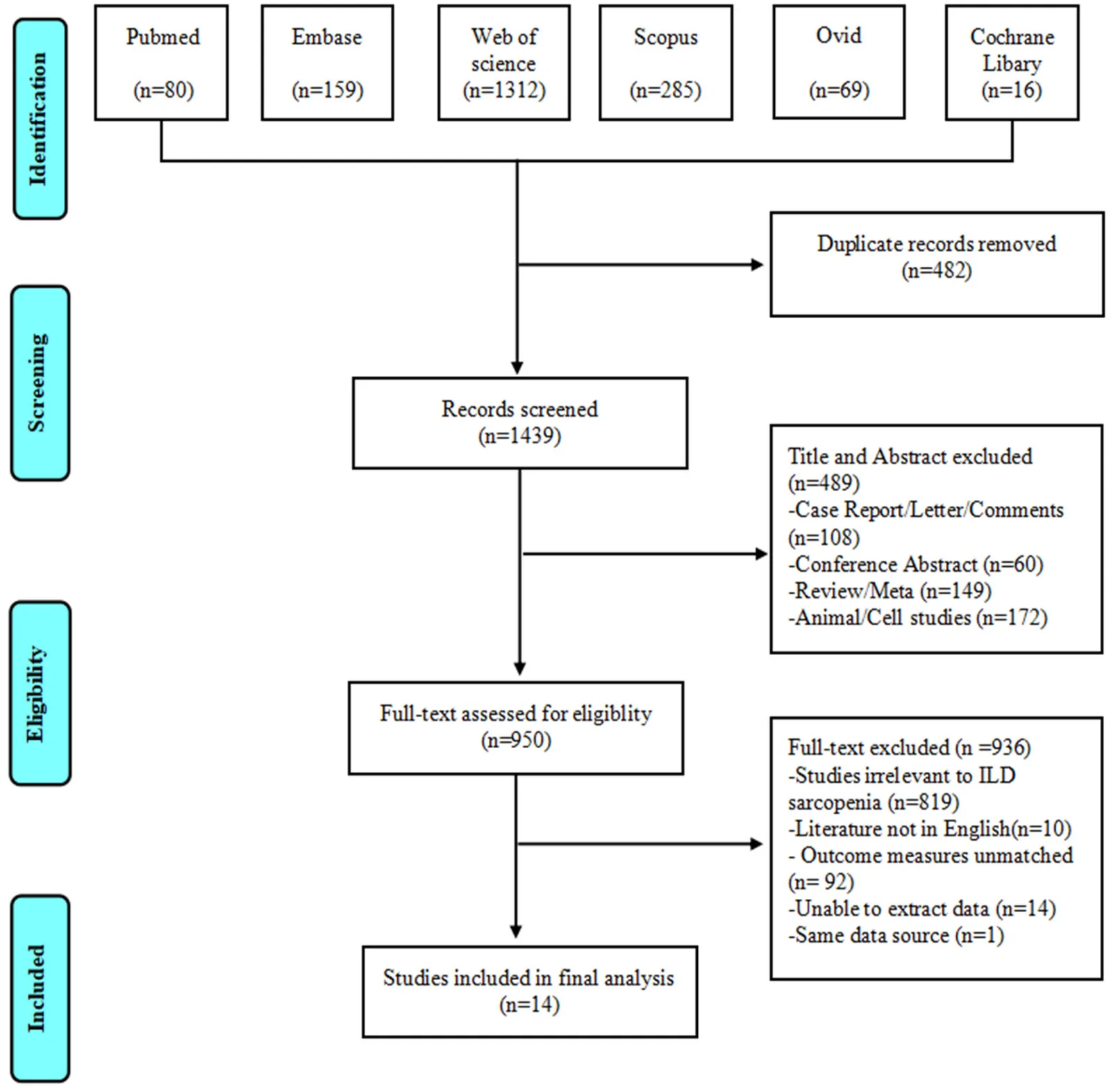
Diagram of the preferred reporting items for systematic review and meta-analysis (PRISMA).

### Difference of age, smoking history and GAP score between ILD and ILD-S

No significant heterogeneity was detected in the overall pooled analyses for age (I^2^ = 0%, *P* = 0.81), smoking history (I^2^ = 0%, *P* = 0.81), and GAP score (I^2^ = 0%, *P* = 0.55). The fixed-effects model (IV) weighting was used for these meta-analyses. The pooled results showed that patients in the ILD-S group were significantly older (WMD = 3.03, 95% CI: 2.12 to 3.93, *P* < 0.001) and had higher GAP scores (WMD = 0.43, 95% CI: 0.15 to 0.71, *P* < 0.001) compared with the ILD group, whereas no significant difference was observed in smoking history (WMD = −1.87, 95% CI: −6.13 to 2.39, *P* = 0.39). Exploratory subgroup analyses were performed according to sarcopenia diagnostic criteria (subgroups 1, 2, and 3) and ILD subtype (subgroups 4 and 5). These analyses are exploratory, and the results are suggested for hypothesis generation. In these exploratory analyses, the increase in age was consistently observed across all subgroups (subgroups 1 to 5, all *P* < 0.05). Elevated GAP scores appeared to be higher in subgroups 2, 3, and 5 (*P* < 0.05), but not in subgroups 1 and 4, each of which included only one study; the direction of effect was consistent. Smoking history showed no notable differences in any subgroup (*P* > 0.05) (Table 1).

**Table 1:** Meta-analysis results of the difference for potential associated variables between ILD and ILD-S.

Variables	Subgroup	No. of study items	No. of	No. of ILD-S	Effects model	I^2^ (%)	*P* value	WMD	*P* value	Egger's test		Metatrim
			total ILD				(heterogeneity)	(95%CI)	(WMD)	t	*P* value	filled study
Age(years)	Subgroup1^a^	3	251	53	Fixed(IV)	0	0.99	3.94 (1.85, 6.02)	0.00	−0.19	0.88	0
	Subgroup2^b^	2**^†^**	134	47	Fixed(IV)	47	0.09	4.56 (1.74, 7.38)	0.00	NA	NA	0
	Subgroup3^c^	5	872	239	Fixed(IV)	0	0.97	2.56 (1.49, 3.64)	0.00	−1.08	0.34	0
	Overall	10	1257	339	Fixed(IV)	0	0.81	3.03 (2.12, 3.93)	0.00	0.08	0.94	0
	Subgroup4^d^	2**^†^**	152	64	Fixed(IV)	0	0.44	2.72 (0.12, 5.31)	0.04	NA	NA	0
	Subgroup5^e^	8	1105	275	Fixed(IV)	0	0.72	3.07 (2.10, 4.03)	0.00	0.37	0.72	0
	Overall	10	1257	339	Fixed(IV)	0	0.81	3.03 (2.12, 3.93)	0.00	0.08	0.94	0
Smoking history(pack-year)	Subgroup2	1**^†^**	56	22	Fixed(IV)	0	NA	−10.53(−24.93, 3.87)	0.15	NA	NA	NA
	Subgroup3	4	798	200	Fixed(IV)	0	0.94	−1.04(−5.501, 3.42)	0.65	−0.93	0.42	0
	Overall (Subgroup5)	5	854	222	Fixed(IV)	0	0.81	−1.87(−6.13, 2.39)	0.39	−1.58	0.19	0
GAP score	Subgroup1	1**^†^**	84	17	Fixed(IV)	NA	NA	−0.97(−3.49, 1.55)	0.45	NA	NA	NA
	Subgroup2	2**^†^**	134	47	Fixed(IV)	0	0.39	0.37 (0.04, 0.69)	0.03	NA	NA	1
	Subgroup3	1^★^**^†^**	180	45	Fixed(IV)	0	0.71	0.66 (0.12, 1.20)	0.02	NA	NA	0
	Overall	4	398	109	Fixed(IV)	0	0.55	0.43 (0.15, 0.71)	0.00	−0.31	0.77	0
	Subgroup4	1**^†^**	78	25	Fixed(IV)	NA	NA	0.29(−0.08, 0.66)	0.13	NA	NA	NA
	Subgroup5	3	320	84	Fixed(IV)	0	0.64	0.61 (0.18, 1.03)	0.01	−3.61	0.07	0
	Overall	4	398	109	Fixed(IV)	0	0.55	0.43 (0.15, 0.71)	0.00	−0.31	0.77	0
FEV_1_% predicted	Subgroup2	2**^†^**	134	47	Fixed(IV)	0	0.58	−4.87(−11.33, 1.60)	0.14	NA	NA	1
	Subgroup3	4	798	200	Fixed(IV)	0	0.46	−7.45(−10.64, −4.26)	0.00	0.44	0.68	1
	Overall	6	932	247	Fixed(IV)	0	0.61	−6.95(−9.80, −4.09)	0.00	0.85	0.43	2
	Subgroup4	1**^†^**	78	25	Fixed(IV)	NA	NA	−2.15(−13.73, 9.43)	0.72	NA	NA	NA
	Subgroup5	5	854	222	Fixed(IV)	0	0.58	−7.26(−10.21, −4.31)	0.00	0.51	0.63	2
	Overall	6	932	247	Fixed(IV)	0	0.61	−6.95(−9.80, −4.09)	0.00	0.85	0.43	2
FVC% predicted	Subgroup1	1**^†^**	83	19	Fixed(IV)	NA	NA	−16.30(−24.14, −8.46)	0.00	NA	NA	NA
	Subgroup2	2**^†^**	134	47	Fixed(IV)	0	0.55	−7.57(−14.10, −1.03)	0.02	NA	NA	1
	Subgroup3	5	871	239	Fixed(IV)	21.4	0.27	−8.86(−11.86, −5.85)	0.00	1.69	0.15	3
	Overall	8	1088	305	Fixed(IV)	21	0.25	−9.46(−12.04, −6.88)	0.00	1.73	0.12	3
	Subgroup4	2**^†^**	152	64	Fixed(IV)	0	0.49	−2.41(−10.16, 5.35)	0.54	NA	NA	1
	Subgroup5	6	936	241	Fixed(IV)	4.5	0.40	−10.34(−13.07, −7.60)	0.00	0.97	0.37	2
	Overall	8	1088	305	Fixed(IV)	21	0.25	−9.46(−12.04, −6.88)	0.00	1.73	0.12	3
FEV_1_/FVC%	Subgroup2	2**^†^**	134	47	Fixed(IV)	0	0.74	1.76(−2.75, 6.28)	0.44	NA	NA	1
	Subgroup3	2**^†^**	375	93	Fixed(IV)	0	0.86	2.01 (0.10, 3.92)	0.04	−0.72	0.55	0
	Overall	4	509	140	Fixed(IV)	0	0.97	1.97 (0.21, 3.73)	0.03	−0.09	0.93	1
	Subgroup4	1**^†^**	78	25	Fixed(IV)	NA	NA	3.94(−9.80, 17.68)	0.57	NA	NA	NA
	Subgroup5	3	431	115	Fixed(IV)	0	0.94	1.94 (0.17, 3.71)	0.03	−0.98	0.40	0
	Overall	4	509	140	Fixed(IV)	0	0.97	1.97 (0.21, 3.73)	0.03	−0.09	0.93	1
DLCO% predicted	Subgroup1	2**^†^**	134	47	Random(DL)	76.6	0.04	−2.58(−17.76, 12.60)	0.74	NA	NA	0
	Subgroup2	2**^†^**	167	36	Random(DL)	17.2	0.27	−2.03(−11.08, 7.02)	0.66	NA	NA	1
	Subgroup3	4	631	168	Random(DL)	58.4	0.05	−8.98(−16.93, −1.03)	0.03	3.04	0.06	2
	Overall	8	932	251	Random(DL)	60.1	0.01	−5.87(−11.68, −0.06)	0.04	3.52	0.01	3
	Subgroup4	2**^†^**	106	41	Random(DL)	0	0.96	4.14(−7.42, 15.71)	0.48	NA	NA	1
	Subgroup5	6	826	210	Random(DL)	60.9	0.02	−7.70 (−13.75, −1.65)	0.01	3.10	0.03	2
	Overall	8	932	251	Random(DL)	60.1	0.01	−5.87(−11.68, −0.06)	0.04	3.52	0.01	3
BMI(kg/m^2^)	Subgroup1	3	251	53	Fixed(IV)	3.7	0.35	−0.11(−1.22, 1.01)	0.85	−2.18	0.27	0
	Subgroup2	2**^†^**	134	47	Fixed(IV)	55.8	0.13	−1.99(−3.32, −0.67)	0.00	NA	NA	1
	Subgroup3	4	566	162	Fixed(IV)	43.4	0.12	−1.65(−2.34, −0.97)	0.00	0.03	0.98	1
	Overall	9	951	262	Fixed(IV)	49	0.03	−1.35(−1.89, −0.82)	0.00	0.73	0.49	0
	Subgroup4	2**^†^**	152	64	Fixed(IV)	0	0.96	−0.34 (−1.70, 1.03)	0.63	NA	NA	1
	Subgroup5	7	799	198	Fixed(IV)	53.2	0.03	−1.54(−2.12, −0.96)	0.00	0.56	0.59	0
	Overall	9	951	262	Fixed(IV)	49	0.03	−1.35(−1.89, −0.82)	0.00	0.73	0.49	0
HGS(kg)	Subgroup1	2**^†^**	168	34	Random(DL)	0	0.90	−16.24(−18.28, −14.21)	0.00	NA	NA	1
	Subgroup2	1^★†^	56	22	Random(DL)	84.6	0.01	−8.71(−16.53, −0.88)	0.03	NA	NA	1
	Overall (Subgroup5)	3	224	56	Random(DL)	83.8	0.00	−12.80(−17.02, −8.58)	0.00	6.40	0.02	0
6MWD(m)	Subgroup1	2**^†^**	167	36	Fixed(IV)	0	0.87	−30.420(−62.61, 1.76)	0.06	NA	NA	1
	Subgroup2	1**^†^**	78	25	Fixed(IV)	NA	NA	−33.60(−100.95,33.75)	0.33	NA	NA	NA
	Overall	3	245	61	Fixed(IV)	0	0.98	−31.01(−60.05, −1.97)	0.04	0.13	0.92	0
	Subgroup4	1**^†^**	167	36	Fixed(IV)	NA	NA	−33.60 (100.95,33.752)	0.33	NA	NA	NA
	Subgroup5	2**^†^**	78	25	Fixed(IV)	0	0.87	−30.42(−62.61, 1.76)	0.06	NA	NA	1
	Overall	3	245	61	Fixed(IV)	0	0.98	−31.01(−60.05, −1.972)	0.04	0.13	0.92	0
ASMI(kg/m^2^)	Subgroup1	2**^†^**	168	34	Random(DL)	97	0.00	−2.492(−5.754, 0.77)	0.13	NA	NA	0
	Subgroup2	1^★^**^†^**	56	22	Random(DL)	84.9	0.01	−0.904(−1.53, −0.279)	0.01	NA	NA	1
	Overall (Subgroup5)	3	224	56	Random(DL)	92.6	0.00	−1.45(−2.145, −0.755)	0.00	−1.14	0.37	0

① a-e: Subgroup analyses were stratified by sarcopenia diagnostic criteria (a-c: Subgroup 1(a): EWGSOP2, Subgroup 2(b): AWGS 2019, Subgroup 3(c): CT imaging-based quantification of skeletal muscle) and adverse outcome follow-up duration (d-e: Subgroup 4(d): ILD patients; Subgroup 5(e): IPF patients). ILD, interstitial lung disease; WMD, weighted mean difference; IV, inverse-variance; GAP, gender, age and physiology; FEV1, forced expiratory volume in one second; FVC, forced vital capacity; DLCO, diffusing capacity of the lungs for carbon monoxide; BMI, body mass index; HGS, Handgrip strength; 6MWD, 6-minute walk distance; ASMI, appendicular skeletal muscle index; NA, not acquired. ② 1★: For a single factor in the same literature, data is extracted hierarchically by gender and other factors, and there will be no situation of repeated calculation. ③ †: This is an exploratory subgroup analysis, and the results need to be interpreted with caution.

### Difference of FEV_1_%, FVC%, FEV_1_/FVC% and DLCO% between ILD and ILD-S

In the overall pooled analyses for FEV1% (I^2^ = 0%, *P* = 0.61), FVC% (I^2^ = 21%, *P* = 0.25), and FEV1/FVC% (I^2^ = 0%, *P* = 0.97), no significant heterogeneity was detected, and fixed-effects model (IV) were applied. For DLCO% (I^2^ = 60.1%, *P* = 0.01), a random-effects model was used. The pooled results demonstrated that the ILD-S group had significantly lower FEV1% (WMD = −6.95, 95% CI: −9.80 to −4.09, *P* < 0.001) and FVC% (WMD = −9.46, 95% CI: −12.04 to −6.88, *P* < 0.001), and a slightly higher FEV1/FVC% ratio (WMD = 1.97, 95% CI: 0.21 to 3.73, *P* = 0.03) compared with the ILD group. The reduction in DLCO% was of borderline significance (WMD = −5.87, 95% CI: −11.68 to −0.06, *P* = 0.04). Exploratory subgroup analyses were largely consistent with the overall findings. The decrease in FEV1% predicted was observed in subgroups 3 and 5 (*P* < 0.05); the decrease in FVC% predicted was observed in subgroups 1, 2, 3, and 5; the increase in FEV1/FVC% was observed in subgroups 3 and 5; and the decrease in DLCO% predicted was observed in subgroups 3 and 5 (Table 1).

### Difference of BMI, HGS, 6MWD and ASMI between ILD and ILD-S

For the overall pooled analyses of BMI (I^2^ = 49%, *P* = 0.03) and 6MWD (I^2^ = 0%, *P* = 0.98), fixed-effects models (IV) weighting were used. For HGS (I^2^ = 83.8%, *P* < 0.001) and ASMI (I^2^ = 92.6%, *P* < 0.001), which showed substantial heterogeneity, random-effects model was used. The ILD-S group had lower BMI (WMD = −1.35, 95% CI: −1.89 to −0.82, *P* < 0.001), HGS (WMD = −12.80, 95% CI: −17.02 to −8.58, *P* < 0.001), 6MWD (WMD = −31.01, 95% CI: −60.05 to −1.97, *P* = 0.04), and ASMI (WMD = −1.45, 95% CI: −2.15 to −0.76, *P* < 0.001) compared with the ILD group. Exploratory subgroup analyses suggested that the reduction in BMI was observed in subgroups 2 and 5; the reduction in HGS was observed in subgroups 1 and 2; and the reduction in ASMI was observed in subgroup 2. The decrease in 6MWD was of borderline significance in the overall analysis and in subgroup 5, but the overall pooled effect was statistically significant. These subgroup findings are exploratory and should be interpreted with caution (Table 1).

### Impact of sarcopenia on mortality hazard ratio in ILD

Eight studies reported on mortality risk, with no significant heterogeneity detected across studies (I^2^ = 0%, *P* = 0.60). A fixed-effects model (IV) was used. The pooled results showed that the ILD-S group had a significantly higher mortality risk than the ILD group (HR = 2.02, 95% CI: 1.67 to 2.45, *P* < 0.001) (Table 2, Figures 2, 3). Exploratory subgroup analyses according to sarcopenia diagnostic criteria suggested that the elevated mortality risk in the ILD-S group was consistently observed in subgroup 1 (HR = 2.18, 95% CI: 1.59 to 3.00, *P* < 0.001), subgroup 2 (HR = 2.17, 95% CI: 1.44 to 3.28, *P* < 0.001), and subgroup 3 (HR = 1.82, 95% CI: 1.34 to 2.46, *P* < 0.001) (Table 2, Figure 2). Exploratory subgroup analyses based on follow-up duration indicated that sarcopenia was associated with mortality risk in both the ≤1-year and >1-year periods (≤1 year: HR = 2.55, 95% CI: 1.55 to 4.19, *P* < 0.001; >1 year: HR = 1.94, 95% CI: 1.57 to 2.39, *P* < 0.001) (Figure 3).

**Table 2:** Meta-analysis results of the hazard ratio for mortality of ILD-S.

Variables	No. of studies	No. of ILD-S	Effects model	I**^2^** (%)	*P* value	HR	*P* value	Egger's test		Metatrim
					(heterogeneity)	(95%CI)	(HR)	t	*P* value	filled study
Mortality										
Subgroup1^a^	2**^†^**	115	Fixed(IV)	0	0.50	2.18 (1.59, 3.00)	0	3.7	0.07	2
Subgroup2^b^	2**^†^**	127	Fixed(IV)	65.9	0.09	2.17 (1.44, 3.28)	0	NA	NA	0
Subgroup3^c^	4	219	Fixed(IV)	0	0.75	1.82 (1.34, 2.46)	0	0.30	0.79	1
Overall	8	461	Fixed(IV)	0	0.60	2.02 (1.67, 2.45)	0	1.16	0.28	3
Subgroup4^d^	1**^†^**	98	Fixed(IV)	NA	NA	1.90 (1.28, 2.81)	0	NA	NA	NA
Subgroup5^e^	7	363	Fixed(IV)	0	0.52	2.06 (1.65, 2.58)	0	1.03	0.34	1
Overall	8	461	Fixed(IV)	0	0.60	2.02 (1.67, 2.45)	0	1.16	0.28	3

① a-e: Subgroup analyses were stratified by sarcopenia diagnostic criteria (a-c: Subgroup 1(a): EWGSOP2, Subgroup 2(b): AWGS 2019, Subgroup 3(c): CT imaging-based quantification of skeletal muscle) and adverse outcome follow-up duration(d-e: Subgroup 4(d): ILD patients; Subgroup 5(e): IPF patients). ILD, interstitial lung disease; HR, hazard ratio; IV, inverse-variance; NA, not acquired. ② †: This is an exploratory subgroup analysis, and the results need to be interpreted with caution.

**Figure 2:**
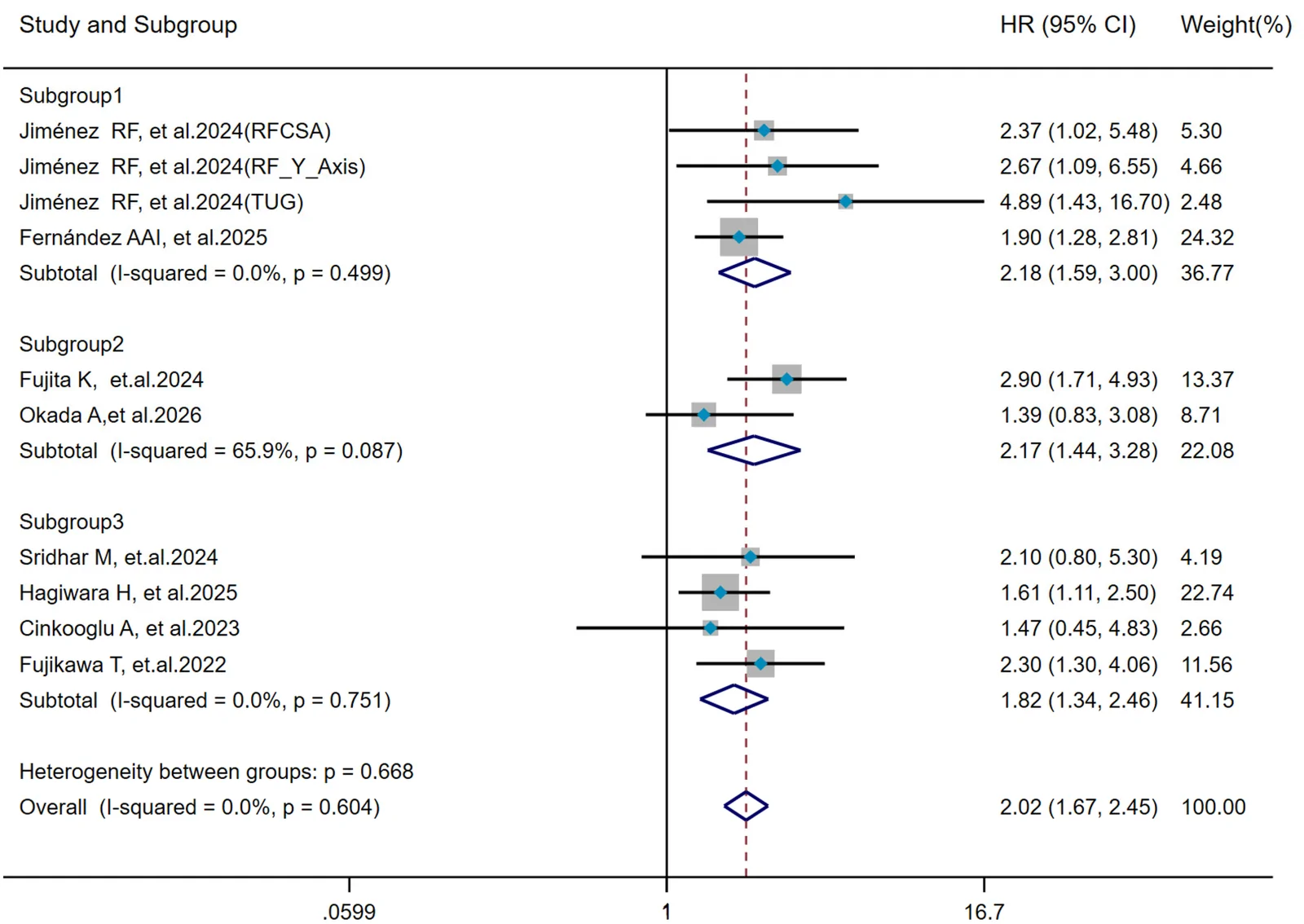
Forest plot of subgroup analyses assessing sarcopenia-associated mortality risk in ILD patients stratified by diagnostic criteria (Subgroup1: EWGSOP2; Subgroup2: AWGS 2019; Subgroup3: CT-based quantification).

**Figure 3:**
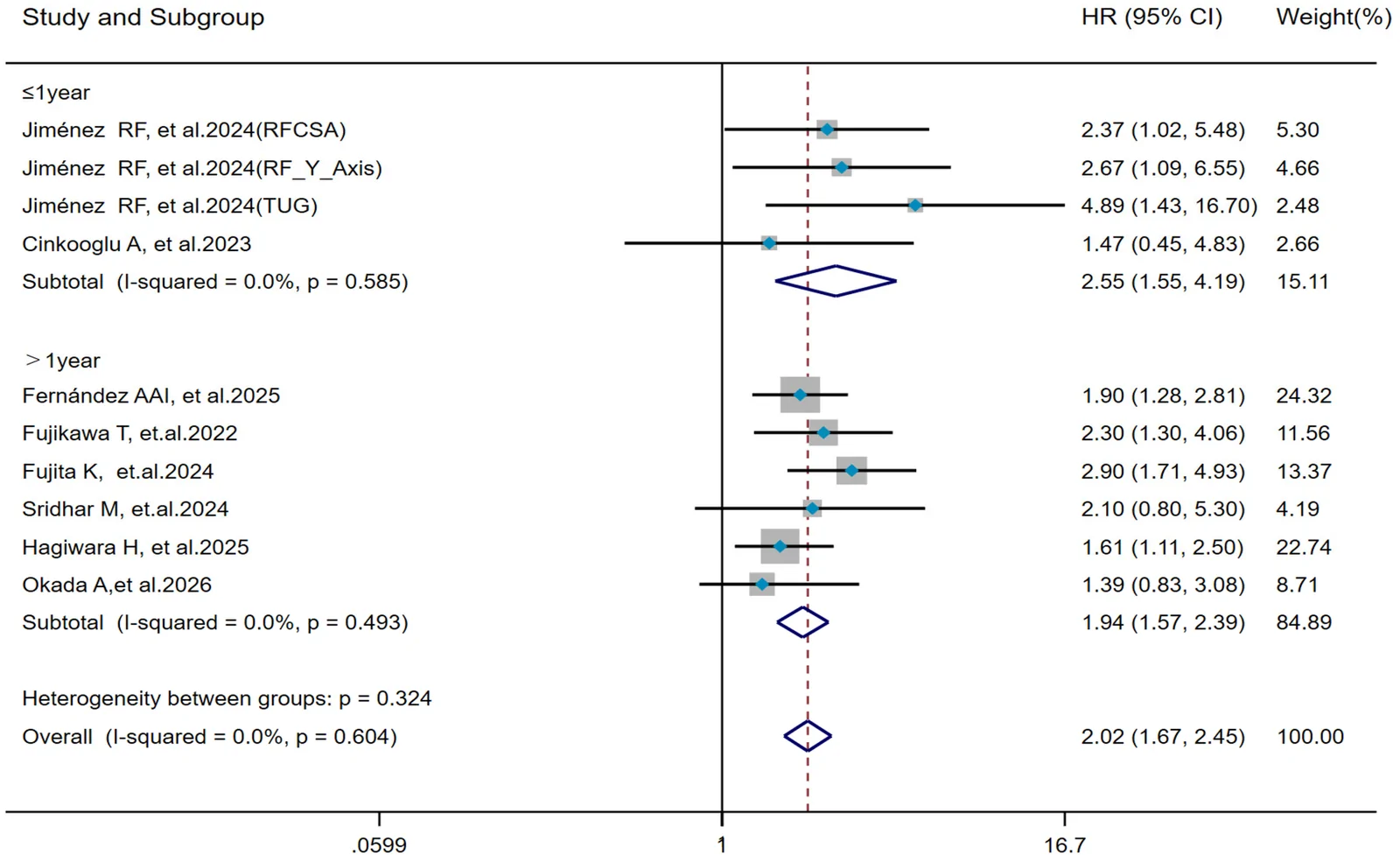
Forest plot of subgroup analyses evaluating sarcopenia-associated mortality risk in ILD patients stratified by follow-up duration (≤1 year; >1 year).

### Sensitivity analysis and publication bias

Sensitivity analysis using the leave-one-out method confirmed the robustness of our pooled results (Supplementary Figures S1–S12). Egger's test suggested possible publication bias for DLCO% and HGS. The trim-and-fill correction estimated the number of potentially missing studies as follows: FEV1% predicted (*n* = 2), FVC% predicted (*n* = 3), FEV1/FVC% (*n* = 1), DLCO% predicted (*n* = 3), and mortality (*n* = 3). After correction, the funnel plots became symmetrical, and the direction and statistical significance of the effect estimates remained unchanged, indicating robust findings (Figures 4A–L).

**Figure 4:**
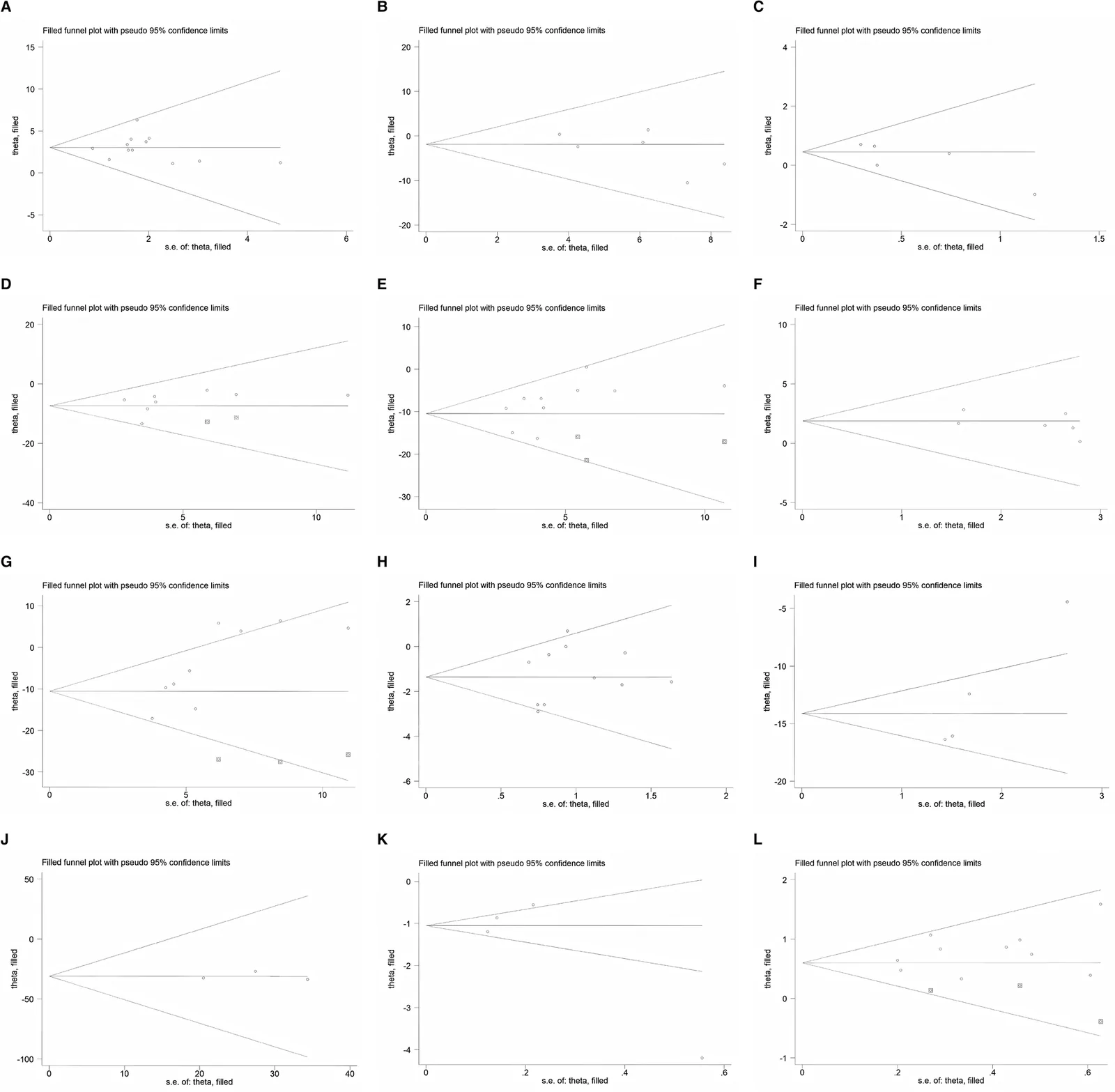
**(A–L)** trim-and-fill method funnel plots for publication bias assessment (A: Age; B: smoking history; C: GAP score; D: FEV_1_%; E: FVC%; F: FEV_1_/FVC%; G: DLCO%; H: BMI; I: HGS; J: 6MWD; K: ASMI; L: mortality).

## Discussion

In recent years, investigating the clinical characteristics and prognosis of ILD-S patients has garnered sustained attention. ILD and sarcopenia exhibit potential bidirectional interactions, posing challenges for patient intervention and disease management. Therefore, clarifying the disease characteristics and mortality risk of ILD-S is crucial for a systematic and comprehensive understanding of the disease, aiding in the development of targeted intervention and management strategies. However, reliable evidence supporting analyses of ILD-S clinical features and poor outcomes is currently lacking. Consequently, we conducted the first pooled analysis to summarize clinical factors associated with ILD complicated by sarcopenia and explore the impact of sarcopenia on poor outcomes in ILD.

Our pooled analysis revealed that ILD-S patients were significantly older than ILD patients. Sarcopenia is closely linked to aging, as the physiological process of muscle atrophy involves a significant association between advancing age and the progressive decline in muscle mass and strength. Chronic reduction and atrophy of muscle tissue substantially increase the risk of sarcopenia. Consequently, the prevalence of ILD-S shows an increasing trend with age. While some studies suggest that smoking directly damages muscle tissue, increasing susceptibility to atrophy and dysfunction and thereby elevating the risk of ILD-S (33), our findings differed. Although our results indicated a positive trend between longer smoking history and ILD-S group risk, it was not statistically significant. This lack of significance may stem from the inclusion of IPF patients within our ILD cohort, as a significant proportion of IPF patients have a smoking history (34), potentially diluting the difference between groups.

Furthermore, we observed that ILD-S patients had higher GAP scores. The GAP score (Gender, Age, Physiology score), incorporating FVC% and DLCO%, is a tool for assessing disease severity and prognosis in ILD patients (35). A higher GAP score indicates more severe ILD and a poorer prognosis (36). Additionally, ILD patients experience declining respiratory function, significantly correlated with reductions in FVC% and DLCO% (37). Considering that sarcopenia is more prevalent in the elderly and among males (38), both of which contribute to higher GAP scores, this demographic association likely underpins the observed score elevation. However, exploratory subgroup analysis of GAP scores showed that in subgroup 1 (EWGSOP2 criteria) and subgroup 4 (classified by ILD subtype), there was no statistically significant difference between the two groups, which was not entirely consistent with the overall trend. This discrepancy may be related to the limited number of studies included in these two subgroups. In addition, differences in baseline characteristics of populations across studies may also affect GAP scores, and further studies with larger sample sizes are needed for verification. In contrast, GAP scores in the ILD-S group appeared to be higher than those in the ILD group in subgroups 2 and 5, suggesting a pattern similar to the overall pooled results. Subgroup 3 used CT quantitative assessment of muscle mass. CT, as a key clinical tool for evaluating muscle mass and quantity, can provide precise quantitative data. Especially for patients with idiopathic pulmonary fibrosis (IPF) who cannot undergo pulmonary function testing, CT examination can also be used indirectly to assess the severity of lung function impairment (39, 40). Moreover, several studies have confirmed that the skeletal muscle index at the T10–L5 vertebral level can be used to assess whole-body muscle mass reduction (40). Therefore, establishing diagnostic indicators at the thoracic level holds promise for a one-stop assessment of both pulmonary pathology and sarcopenia. Regarding ILD subtypes, the GAP score is more widely applied in clinical practice for IPF and demonstrates higher diagnostic sensitivity compared to other ILD.

This study found that ILD-S patients had significantly lower FEV1% and FVC%, borderline significantly lower DLCO%, and a slightly higher FEV1/FVC% ratio. This indicates that ILD-S patients have impaired pulmonary function, which may be related to reduced respiratory muscle strength and function (10). In addition, sarcopenia may indicate more severe underlying disease in ILD patients (30), with greater disease burden. Notably, ILD mainly presents as restrictive ventilatory dysfunction, characterized by proportional decline of FEV1 and FVC, or more pronounced decline of FVC, which may explain why the FEV1/FVC% ratio remains normal or even elevated. Exploratory subgroup analyses suggested potential differences according to sarcopenia diagnostic criteria. This variation could stem from multiple factors. As mentioned earlier, CT directly measures muscle mass, whereas DXA/BIA methods used in AWGS2019 and EWGSOP2 estimate lean body mass (excluding fat and bone), making them susceptible to hydration status fluctuations and less accurate for trunk muscle assessment. Consequently, Subgroup 3 (CT-based) may reflect pulmonary function parameters more accurately. Secondly, low muscle strength is a primary diagnostic criterion for sarcopenia, with HGS being the most common measure (41). In ILD patients, systemic inflammation, hypoxemia, and reduced physical activity may lead to decreased muscle strength (lower HGS), and this decline often precedes the deterioration of physical function, potentially resulting in more pronounced worsening of pulmonary function. Compared with AWGS2019, the EWGSOP2 criteria set a stricter threshold for defining low muscle strength. However, comparative studies directly evaluating AWGS2019, EWGSOP2, and CT-based diagnostic criteria are scarce and require further validation in prospective studies.

Our analysis also found that low BMI, low HGS, and low ASMI were factors associated with ILD-S. Zhang et al. (42) identified low BMI as a risk factor for sarcopenia, which is consistent with our findings. ASMI and HGS are key indicators in the AWGS 2019 criteria and the EWGSOP2 criteria, respectively; the former is used to assess appendicular skeletal muscle mass, and the latter is used to assess muscle strength. In addition, the 6MWD in our study reached statistical significance only marginally, and the number of included studies was limited, so more high-quality studies are needed for validation. Although previous studies have reported that reduced 6MWD is an independent risk factor associated with patients with sarcopenia (22), our pooled analysis, due to the limited number of included studies, is insufficient to draw a definitive conclusion. This may be because 6MWD is also influenced by age, exercise tolerance, comorbidities, and disease severity. This association warrants further investigation with larger sample sizes and designs controlling for confounding factors.

Our meta-analysis showed that the mortality risk of ILD-S patients was approximately 2.02 times that of ILD patients, suggesting that sarcopenia is closely associated with elevated mortality risk in ILD patients and may serve as a potential prognostic marker. As the feasibility of clinically assessing sarcopenia-related parameters increases, its importance as a prognostic indicator and potential therapeutic target for ILD patients is gaining more attention (43, 44). For ILD patients, the interaction between underlying lung disease severity and decline in physical function may jointly increase their mortality risk (43). Exploratory subgroup analyses stratified by follow-up duration suggested that the sarcopenia-associated mortality risk appeared to remain relatively consistent across both the ≤1-year and >1-year follow-up periods. These exploratory findings may indicate some predictive value for both short-term and long-term prognosis, and might offer additional support for the potential role of sarcopenia in mortality risk assessment for ILD patients.

Notably, research into ILD-S-related factors and prognosis is an ongoing endeavor, and several critical questions remain. Firstly, although evidence indicates sarcopenia is prevalent in ILD and linked to poorer outcomes, many cohort studies are small and overrepresent male IPF patients. Exploring these relationships in larger, more diverse ILD populations is essential. Secondly, despite diverse sarcopenia assessment methods and established links between low muscle mass and disease severity indices, standardized reference ranges for muscle parameters and a universally accepted gold standard for muscle quality assessment in the ILD population are still lacking. This remains a significant challenge in clinical practice. CT imaging emerges as a promising tool, with evidence suggesting that scans acquired during routine ILD evaluation can be repurposed for muscle mass quantification (23, 24). However, the applicability and clinical value of this approach across different ILD subtypes remain to be established. Several limitations of the present meta-analysis should be acknowledged. The small sample sizes of all included studies mean that the identified risk factors need confirmation in larger, prospective multicenter cohorts. Restricting the search to English-language publications may have introduced language bias. In addition, insufficient data precluded a pooled analysis of several other potential risk factors mentioned in some studies, such as the Timed Up and Go test, SGRQ score, SARC-F score, and measures of anxiety or depression in relation to ILD-S. Finally, because the number of eligible studies was limited, further research is needed to better characterize the clinical features of sarcopenia in each ILD subtype and to explore its relationship with disease progression, infectious complications, exacerbations, and overall mortality. Furthermore, the number of included studies was limited, and some subgroup analyses were exploratory. These exploratory findings are more suitable for hypothesis generation than for confirmatory inference, so further validation in larger and better-designed studies is warranted.

## Conclusions

In summary, our pooled analysis indicates that older age, higher GAP score, decreased pulmonary function, lower BMI, lower HGS, and lower ASMI are potential factors associated with ILD-S. Meanwhile, sarcopenia was found to be significantly associated with increased mortality risk in ILD patients. These findings suggest that sarcopenia-related parameters may have potential value in the prognosis assessment of ILD patients. Early identification of these clinical features in ILD-S and implementing management interventions may become crucial strategies for improving poor outcomes in ILD patients.

## Data Availability

The original contributions presented in the study are included in the article/Supplementary Material, further inquiries can be directed to the corresponding author.
