# Supplementary material for: Clinical characteristics and mortality risk of sarcopenia in interstitial lung disease: a systematic review and meta-analysis
Source: Front Med (Lausanne). 2026 Oct 5;13:1955396. doi: 10.3389/fmed.2026.1955396 (PMC13677835; doi:10.3389/fmed.2026.1955396)
Supplement: Supplementary file 1 [file Supplementaryfile1.docx]

Supplementary Table 1. Database and Search strategy

| **Database** | **Search strategy** |
| --- | --- |
| **PubMed (n=80)** | #1: ((((((((((((((Lung Diseases, Interstitial[MeSH Terms]) OR (Diffuse Parenchymal Lung Diseases[Title/Abstract])) OR (Interstitial Lung Diseases[Title/Abstract])) OR (Interstitial Lung Disease[Title/Abstract])) OR (Lung Disease, Interstitial[Title/Abstract])) OR (Diffuse Parenchymal Lung Disease[Title/Abstract])) OR (Pneumonia, Interstitial[Title/Abstract])) OR (Interstitial Pneumonia[Title/Abstract])) OR (Interstitial Pneumonias[Title/Abstract])) OR (Pneumonias, Interstitial[Title/Abstract])) OR (Pneumonitis, Interstitial[Title/Abstract])) OR (Interstitial Pneumonitides[Title/Abstract])) OR (Interstitial Pneumonitis[Title/Abstract])) OR (Pneumonitides, Interstitial[Title/Abstract])) OR (ILD[Title/Abstract])  N=110691 |
|  | #2: (((((((((((((((((((((Idiopathic Pulmonary Fibrosis[MeSH Terms]) OR (Pulmonary Fibroses, Idiopathic[Title/Abstract])) OR (Cryptogenic Fibrosing Alveolitis[Title/Abstract])) OR (Cryptogenic Fibrosing Alveolitides[Title/Abstract])) OR (Fibrosing Alveolitides, Cryptogenic[Title/Abstract])) OR (Pulmonary Fibrosis, Idiopathic[Title/Abstract])) OR (Fibrosing Alveolitis, Cryptogenic[Title/Abstract])) OR (Fibrocystic Pulmonary Dysplasia[Title/Abstract])) OR (Dysplasia, Fibrocystic Pulmonary[Title/Abstract])) OR (Fibrocystic Pulmonary Dysplasias[Title/Abstract])) OR (Pulmonary Dysplasia, Fibrocystic[Title/Abstract])) OR (Idiopathic Fibrosing Alveolitis, Chronic Form[Title/Abstract])) OR (Familial Idiopathic Pulmonary Fibrosis[Title/Abstract])) OR (Idiopathic Pulmonary Fibrosis, Familial[Title/Abstract])) OR (Usual Interstitial Pneumonia[Title/Abstract])) OR (Interstitial Pneumonia, Usual[Title/Abstract])) OR (Usual Interstitial Pneumonias[Title/Abstract])) OR (Interstitial Pneumonitis, Usual[Title/Abstract])) OR (Pneumonitides, Usual Interstitial[Title/Abstract])) OR (Pneumonitis, Usual Interstitial[Title/Abstract])) OR (Usual Interstitial Pneumonitides[Title/Abstract])) OR (Usual Interstitial Pneumonitis[Title/Abstract])  N=20813 |
|  | #3: #1 OR #2=114438 |
|  | #4: ((((((Sarcopenia[MeSH Terms]) OR (Sarcopenias[Title/Abstract])) OR (Muscle atrophy[Title/Abstract])) OR (Sarcopenic[Title/Abstract])) OR (Muscle attenuation[Title/Abstract])) OR (Muscle loss[Title/Abstract])) OR (Muscle depletion[Title/Abstract])  N=34200 |
|  | #5: #3 AND #4 = **80** items |
| **Embase (n=159)** | #1: 'Lung Diseases, Interstitial'/exp OR 'Diffuse Parenchymal Lung Diseases':ab,ti,kw OR 'Interstitial Lung Diseases':ab,ti,kw OR 'Interstitial Lung Disease':ab,ti,kw OR 'Lung Disease, Interstitial':ab,ti,kw OR 'Diffuse Parenchymal Lung Disease':ab,ti,kw OR 'Pneumonia, Interstitial':ab,ti,kw OR 'Interstitial Pneumonia':ab,ti,kw OR 'Interstitial Pneumonias':ab,ti,kw OR 'Pneumonias, Interstitial':ab,ti,kw OR 'Pneumonitis, Interstitial':ab,ti,kw OR 'Interstitial Pneumonitides':ab,ti,kw OR 'Interstitial Pneumonitis':ab,ti,kw OR 'Pneumonitides, Interstitial':ab,ti,kw OR 'ILD':ab,ti,kw  N=65111 |
|  | #2: 'idiopathic pulmonary fibrosis'/exp OR 'Pulmonary Fibroses, Idiopathic':ab,ti,kw OR 'Cryptogenic Fibrosing Alveolitis':ab,ti,kw OR 'Cryptogenic Fibrosing Alveolitides':ab,ti,kw OR 'Fibrosing Alveolitides, Cryptogenic':ab,ti,kw OR 'Pulmonary Fibrosis, Idiopathic':ab,ti,kw OR 'Fibrosing Alveolitis, Cryptogenic':ab,ti,kw OR 'Fibrocystic Pulmonary Dysplasia':ab,ti,kw OR 'Dysplasia, Fibrocystic Pulmonary':ab,ti,kw OR 'Fibrocystic Pulmonary Dysplasias':ab,ti,kw OR 'Pulmonary Dysplasia, Fibrocystic':ab,ti,kw OR 'Idiopathic Fibrosing Alveolitis, Chronic Form':ab,ti,kw OR 'Familial Idiopathic Pulmonary Fibrosis':ab,ti,kw OR 'Idiopathic Pulmonary Fibrosis, Familial':ab,ti,kw OR 'Usual Interstitial Pneumonia':ab,ti,kw OR 'Interstitial Pneumonia, Usual':ab,ti,kw OR 'Usual Interstitial Pneumonias':ab,ti,kw OR 'Interstitial Pneumonitis, Usual':ab,ti,kw OR 'Pneumonitides, Usual Interstitial':ab,ti,kw OR 'Pneumonitis, Usual Interstitial':ab,ti,kw OR 'Usual Interstitial Pneumonitides':ab,ti,kw OR 'Usual Interstitial Pneumonitis':ab,ti,kw  N=4748 |
|  | #3 :#1 OR #2= 65447 |
|  | 'Sarcopenia':ab,ti,kw OR 'Sarcopenias':ab,ti,kw OR 'Muscle atrophy':ab,ti,kw OR 'Sarcopenic':ab,ti,kw OR 'Muscle attenuation':ab,ti,kw OR 'Muscle loss':ab,ti,kw OR 'Muscle depletion':ab,ti,kw |
|  | #5: #3 AND #4 = **159** items |
| **Cochrane library**  **(n=16)** | #1: (Lung Diseases, Interstitial):MeSH OR (Interstitial Lung Diseases):ab,ti,kw OR (Interstitial Lung Disease):ab,ti,kw OR (Lung Disease, Interstitial):ab,ti,kw OR (Diffuse Parenchymal Lung Disease):ab,ti,kw OR (Pneumonia, Interstitial):ab,ti,kw OR (Interstitial Pneumonia):ab,ti,kw OR (Interstitial Pneumonias):ab,ti,kw OR (Pneumonias, Interstitial):ab,ti,kw OR (Pneumonitis, Interstitial):ab,ti,kw OR (Interstitial Pneumonitides):ab,ti,kw OR (Interstitial Pneumonitis):ab,ti,kw OR (Pneumonitides, Interstitial):ab,ti,kw OR (ILD):ab,ti,kw  N=3389 |
|  | #2: (Idiopathic Pulmonary Fibrosis):MeSH OR (Cryptogenic Fibrosing Alveolitis):ab,ti,kw OR (Cryptogenic Fibrosing Alveolitides):ab,ti,kw OR (Fibrosing Alveolitides, Cryptogenic):ab,ti,kw OR (Pulmonary Fibrosis, Idiopathic):ab,ti,kw OR (Fibrosing Alveolitis, Cryptogenic):ab,ti,kw OR (Fibrocystic Pulmonary Dysplasia):ab,ti,kw OR (Dysplasia, Fibrocystic Pulmonary):ab,ti,kw OR (Fibrocystic Pulmonary Dysplasias):ab,ti,kw OR (Pulmonary Dysplasia, Fibrocystic):ab,ti,kw OR (Idiopathic Fibrosing Alveolitis, Chronic Form):ab,ti,kw OR (Familial Idiopathic Pulmonary Fibrosis):ab,ti,kw OR (Idiopathic Pulmonary Fibrosis, Familial):ab,ti,kw OR (Usual Interstitial Pneumonia):ab,ti,kw OR (Interstitial Pneumonia, Usual):ab,ti,kw  N=1800 |
|  | #3:#1 OR #2=4721 |
|  | #4: (Sarcopenia):MeSH OR (Sarcopenic):ab,ti,kw OR (Muscle attenuation):ab,ti,kw OR (Muscle loss):ab,ti,kw OR (Muscle depletion):ab,ti,kw  N=12808 |
|  | #5:#3 AND #4 = **16** items |
| **Web of science**  **(n=1312)** | #1: Lung Diseases, Interstitial OR Diffuse Parenchymal Lung Diseases OR Interstitial Lung Diseases OR Interstitial Lung Disease OR Lung Disease, Interstitial OR Diffuse Parenchymal Lung Disease OR Pneumonia, Interstitial OR Interstitial Pneumonia OR Interstitial Pneumonias OR Pneumonias, Interstitial OR Pneumonitis, Interstitial OR Interstitial Pneumonitides OR Interstitial Pneumonitis OR Pneumonitides, Interstitial OR ILD  N= 84121 |
|  | #2: Idiopathic Pulmonary Fibrosis OR Pulmonary Fibroses, Idiopathic OR Cryptogenic Fibrosing Alveolitis OR Cryptogenic Fibrosing Alveolitides OR Fibrosing Alveolitides, Cryptogenic OR Pulmonary Fibrosis, Idiopathic OR Fibrosing Alveolitis, Cryptogenic OR Fibrocystic Pulmonary Dysplasia OR Dysplasia, Fibrocystic Pulmonary OR Fibrocystic Pulmonary Dysplasias OR Pulmonary Dysplasia, Fibrocystic OR Idiopathic Fibrosing Alveolitis, Chronic Form OR Familial Idiopathic Pulmonary Fibrosis OR Idiopathic Pulmonary Fibrosis, Familial OR Usual Interstitial Pneumonia OR Interstitial Pneumonia, Usual OR Usual Interstitial Pneumonias OR Interstitial Pneumonitis, Usual OR Pneumonitides, Usual Interstitial OR Pneumonitis, Usual Interstitia OR Usual Interstiti al Pneumonitides OR Usual Interstitial Pneumonitis  N= 41612 |
|  | #3:#1 OR #2=117723 |
|  | #4: Sarcopenia OR Sarcopenias OR Muscle atrophy OR Sarcopenic OR Muscle attenuation OR Muscle loss OR Muscle depletion  N=270887 |
|  | #5:#3AND #4 =**1312 items** |
| **Scopus**  **(n=285)** | #1: TITLE-ABS-KEY ("Diffuse Parenchymal Lung Diseases" OR "Interstitial Lung Diseases" OR "Interstitial Lung Disease" OR "Lung Disease, Interstitial" OR "Diffuse Parenchymal Lung Disease" OR "Pneumonia, Interstitial" OR "Interstitial Pneumonia" OR "Interstitial Pneumonias" OR "Pneumonias, Interstitial" OR "Pneumonitis, Interstitial" OR "Interstitial Pneumonitides" OR "Interstitial Pneumonitis" OR "Pneumonitides, Interstitial" OR "ILD")  N=59122 |
|  | #2: TITLE-ABS-KEY ( "Idiopathic Pulmonary Fibrosis" OR "Pulmonary Fibroses, Idiopathic" OR "Cryptogenic Fibrosing Alveolitis" OR "Cryptogenic Fibrosing Alveolitides" OR "Fibrosing Alveolitides, Cryptogenic" OR "Pulmonary Fibrosis, Idiopathic" OR "Fibrosing Alveolitis, Cryptogenic" OR "Fibrocystic Pulmonary Dysplasia" OR "Dysplasia, Fibrocystic Pulmonary" OR "Fibrocystic Pulmonary Dysplasias" OR "Pulmonary Dysplasia, Fibrocystic" OR "Idiopathic Fibrosing Alveolitis, Chronic Form" OR "Familial Idiopathic Pulmonary Fibrosis" OR "Idiopathic Pulmonary Fibrosis, Familial" OR "Usual Interstitial Pneumonia" OR "Interstitial Pneumonia, Usual" OR "Usual Interstitial Pneumonias" OR "Interstitial Pneumonitis, Usual" OR "Pneumonitides, Usual Interstitial" OR "Pneumonitis, Usual Interstitia" OR "Usual Interstitial Pneumonitides" OR "Usual Interstitial Pneumonitis")  N=18532 |
|  | #3:#1 OR #2=68810 |
|  | #4: TITLE-ABS-KEY ("Sarcopenia" OR "Sarcopenias" OR "Muscle atrophy" OR "Sarcopenic" OR "Muscle attenuation" OR "Muscle loss" OR "Muscle depletion")  N=75347 |
|  | #5:#4 AND #3 =**285** items |
| **Ovid**  **(n=69)** | #1: ( Lung Diseases, Interstitial or Diffuse Parenchymal Lung Diseases or Interstitial Lung Diseases or Interstitial Lung Disease or Lung Disease, Interstitial or Diffuse Parenchymal Lung Disease or Pneumonia, Interstitial or Interstitial Pneumonia or Interstitial Pneumonias or Pneumonias, Interstitial or Pneumonitis, Interstitial or Interstitial Pneumonitides or Interstitial Pneumonitis or Pneumonitides, Interstitial or ILD ).ti,ab,kw.  N=30890 |
|  | #2:(Idiopathic Pulmonary Fibrosis or Pulmonary Fibroses, Idiopathic or Cryptogenic Fibrosing Alveolitis or Cryptogenic Fibrosing Alveolitides or Fibrosing Alveolitides, Cryptogenic or Pulmonary Fibrosis, Idiopathic or Fibrosing Alveolitis, Cryptogenic or Fibrocystic Pulmonary Dysplasia or Dysplasia, Fibrocystic Pulmonary or Fibrocystic Pulmonary Dysplasias or Pulmonary Dysplasia, Fibrocystic or Idiopathic Fibrosing Alveolitis, Chronic Form or Familial Idiopathic Pulmonary Fibrosis or Idiopathic Pulmonary Fibrosis, Familial).ti,ab,kw.  N=16712 |
|  | #3:#1 or #2=31171 |
|  | #4: (Sarcopenia or Sarcopenias or Muscle atrophy or Sarcopenic or Muscle attenuation or Muscle loss or Muscle depletion).ti,ab,kw  N=35771 |
|  | #5:#3 AND #4 =**69** items |

Supplementary Table 2 Results of Newcastle-Ottawa quality assessment Scale for each included study

| **Study** | **Selection** | **Comparability** | **Outcome** | **Total** |
| --- | --- | --- | --- | --- |
| Fernández-Jiménez R, et al.2024 | ★★★★ | ★★ | ★★ | 8★ |
| Fujita K, et al.2022 | ★★★★ | ★★ | ★★ | 8★ |
| Faverio P, et al.2022 | ★★★★ | ★★ | ★★ | 8★ |
| Moon SW, et al.2019 | ★★★★ | ★★ | ★★ | 8★ |
| Cinkooglu A, et al.2023 | ★★★★ | ★ | ★★ | 7★ |
| Fujikawa T, et al.2022 | ★★★★ | ★ | ★★★ | 8★ |
| Hanada M, et al.2022 | ★★★★ | ★ | ★★ | 7★ |
| Kikuchi R, et al.2021 | ★★★★ | ★ | ★★ | 7★ |
| Hagiwara H, et al.2025 | ★★★★ | ★★ | ★★★ | 9★ |
| Cabrera-César E, et al.2025 | ★★★★ | ★ | ★★ | 7★ |
| Fujita K, et.al.2024 | ★★★★ | ★★ | ★★★ | 9★ |
| Ibarra-Fernández AA, et al.2025 | ★★★★ | ★★ | ★★ | 8★ |
| Sridhar M, et.al.2024 | ★★★★ | ★★ | ★★ | 8★ |
| Okada A,et al.2026 | ★★★★ | ★★ | ★★★ | 9★ |

Supplementary Table 3 Characteristics of each included study

| **No** | **Author** | **Time** | **Country** | **Study type** | **ILD type** | **Total ILD** | **Comparison group information** | | | | | **Cox regression analysis** | | | **Survival time of Cox regression analysis (month)** | **Sarcopenia diagnostic criteria** |
| --- | --- | --- | --- | --- | --- | --- | --- | --- | --- | --- | --- | --- | --- | --- | --- | --- |
|  |  |  |  |  |  | **(n)** | **Variable** | **ILD group** | | **ILD-S group** | |  |  |  |  |  |
|  |  |  |  |  |  |  |  | **n** | **mean ± SD** | **n** | **mean ± SD** | **HR** | **Lower limit** | **Higher limit** |  |  |
| 1 | Fernández-Jiménez R, et al[29] | 2024 | Spain | prospective | IPF | 84 | Age(year) | 67 | 70.2 ± 7.1 | 17 | 74.3 ± 7.5 | 2.37^a^ | 1.02 | 5.48 | 12 | EWGSOP2 |
|  |  |  |  |  |  |  | BMI(kg/m^2^) |  | 27.5 ± 3.46 |  | 28.2 ± 3.47 | 2.67^b^ | 1.09 | 6.55 |  |  |
|  |  |  |  |  |  |  | GAP index^*^ |  | 4.35 ± 5.3 |  | 3.38 ± 4.59 |  |  |  |  |  |
|  |  |  |  |  |  |  | HGS(kg) |  | 36.3 ± 8.27 |  | 20.2 ± 4.61 | 4.89^c^ | 1.43 | 16.7 |  |  |
|  |  |  |  |  |  |  | ASMI(kg/m^2^) |  | 21.3 ± 3.11 |  | 17.1 ± 1.67 |  |  |  |  |  |
| 2 | Fujita K, et al[22] | 2022 | Japan | prospective | IPF | 56 | Age(year) | 34 | 70.6 ± 8.1 | 22 | 76.9 ± 5.1 | NA | NA | NA | NA | AWGS 2019 |
|  |  |  |  |  |  |  | BMI(kg/m^2^) |  | 23.3 ± 3.0 |  | 20.7 ± 2.8 |  |  |  |  |  |
|  |  |  |  |  |  |  | Smoking history(package/year) |  | 34.09 ± 24 |  | 23.28 ± 28.53 |  |  |  |  |  |
|  |  |  |  |  |  |  | GAP index^*^ |  | 3.36 ± 0.77 |  | 4 ± 1.59 |  |  |  |  |  |
|  |  |  |  |  |  |  | FEV_1_% |  | 83.8 ± 15.6 |  | 77.7 ± 13.8 |  |  |  |  |  |
|  |  |  |  |  |  |  | FVC% |  | 83.7 ± 16.3 |  | 74.6 ± 14.8 |  |  |  |  |  |
|  |  |  |  |  |  |  | FEV_1_/FVC% |  | 81 ± 8.3 |  | 82.5 ± 9.3 |  |  |  |  |  |
|  |  |  |  |  |  |  | DLCO% |  | 68.5 ± 18 |  | 62.9 ± 19.2 |  |  |  |  |  |
|  |  |  |  |  |  |  | ASMI(male)^*^(kg/m^2^) | 30 | 36.92 ± 5.99 | 19 | 25.85 ± 4.41 |  |  |  |  |  |
|  |  |  |  |  |  |  | ASMI(female)^*^(kg/m^2^) | 4 | 19.66 ± 3.71 | 3 | 15.22 ± 3.29 |  |  |  |  |  |
|  |  |  |  |  |  |  | HGS(male)^*^(kg) | 30 | 36.92 ± 5.99 | 19 | 24.48 ± 5.53 |  |  |  |  |  |
|  |  |  |  |  |  |  | HGS(female)^*^(kg) | 4 | 19.66 ± 3.71 | 3 | 15.22 ± 3.29 |  |  |  |  |  |
| 3 | Faverio P, et al[30] | 2022 | Italy | prospective | IPF | 83 | Age(year) | 64 | 71.6 ± 6.9 | 19 | 75.6 ± 6.9 | NA | NA | NA | NA | EWGSOP2 |
|  |  |  |  |  |  |  | BMI(kg/m^2^) |  | 28.0 ± 3.9 |  | 26.6 ± 4.4 |  |  |  |  |  |
|  |  |  |  |  |  |  | FVC% |  | 89.5 ± 22.1 |  | 73.2 ± 12.6 |  |  |  |  |  |
|  |  |  |  |  |  |  | DLCO% |  | 56.5 ± 19.0 |  | 46.8 ± 15.4 |  |  |  |  |  |
|  |  |  |  |  |  |  | 6MWD(m) |  | 409.4 ± 114.0 |  | 382.6 ± 102.0 |  |  |  |  |  |
| 4 | Kikuchi R, et al[24] | 2021 | Japan | retrospective | DI-ILD | 74 | Age^*^(year) | 35 | 69 ± 7.73 | 39 | 72.36 ± 5.39 | NA | NA | NA | NA | psoas muscle index cutoff values for sarcopenia |
|  |  |  |  |  |  |  | BMI^*^(kg/m^2^) |  | 23.11 ± 4.25 |  | 22.75 ± 2.46 |  |  |  |  |  |
|  |  |  |  |  |  |  | FVC%^*^ |  | 100.87 ± 21.88 |  | 101.37 ± 27.55 |  |  |  |  |  |
|  |  |  |  |  |  |  | DLCO%^*^ | 12 | 66.08 ± 35.72 | 16 | 70.72 ± 14.63 |  |  |  |  |  |
| 5 | Moon SW, et al[31] | 2019 | South Korea | retrospective | IPF | 180 | Age(male)(year) | 107 | 68.1 ± 7.9 | 36 | 70.8 ± 8.9 | NA | NA | NA | NA | Lowest quartile of middle of 4th or 12th thoracic vertebrae muscle index (T4MI) or (T12MI) in male and female respectively. |
|  |  |  |  |  |  |  | Age(female)(year) | 28 | 70.4 ± 8.2 | 9 | 71.6 ± 13.2 |  |  |  |  |  |
|  |  |  |  |  |  |  | BMI(male)(kg/m^2^) | 107 | 24 ± 3.1 | 36 | 23.3 ± 3.7 |  |  |  |  |  |
|  |  |  |  |  |  |  | BMI(female)(kg/m^2^) | 28 | 24.4 ± 3.1 | 9 | 22.7 ± 3.5 |  |  |  |  |  |
|  |  |  |  |  |  |  | Smoking history(male) (package/year) | 107 | 32.1 ± 23.9 | 36 | 29.7 ± 21.5 |  |  |  |  |  |
|  |  |  |  |  |  |  | Smoking history(female) (package/year) | 28 | 8.3 ± 7.6 | 9 | 0 |  |  |  |  |  |
|  |  |  |  |  |  |  | GAP index(male) | 107 | 3.3 ± 1.3 | 36 | 4.0 ± 1.6 |  |  |  |  |  |
|  |  |  |  |  |  |  | GAP index(female) | 28 | 2.7 ± 1.3 | 9 | 3.1 ± 2.1 |  |  |  |  |  |
|  |  |  |  |  |  |  | FEV_1_%(male) | 107 | 90.4 ± 18 | 36 | 86.1 ± 21.2 |  |  |  |  |  |
|  |  |  |  |  |  |  | FEV_1_%(female) | 28 | 88.9 ± 17.8 | 9 | 85.3 ± 18.4 |  |  |  |  |  |
|  |  |  |  |  |  |  | FVC%(male) | 107 | 78.9 ± 16.2 | 36 | 72 ± 18.9 |  |  |  |  |  |
|  |  |  |  |  |  |  | FVC%(female) | 28 | 73.8 ± 16.3 | 9 | 68.7 ± 18.1 |  |  |  |  |  |
|  |  |  |  |  |  |  | FEV_1_/FVC%(male) | 107 | 79.8 ± 9.3 | 36 | 82.6 ± 8.1 |  |  |  |  |  |
|  |  |  |  |  |  |  | FEV_1_/FVC%(female) | 28 | 84.6 ± 7.8 | 9 | 87.1 ± 6.6 |  |  |  |  |  |
|  |  |  |  |  |  |  | DLCO%(male) | 107 | 73.7 ± 22.8 | 36 | 64.9 ± 23.9 |  |  |  |  |  |
|  |  |  |  |  |  |  | DLCO%(female) | 28 | 59.8 ± 20.3 | 9 | 66.2 ± 22.6 |  |  |  |  |  |
| 6 | Cinkooglu A, et al[25] | 2023 | Turkey | retrospective | IPF | 195 | Age(male)(year) | 117 | 68.46± 7.57 | 38 | 70.03 ± 6.02 | 1.899 | 1.282 | 2.811 | 12 | lowest quartile of erector spinae muscle area |
|  |  |  |  |  |  |  | Age(female)(year) | 30 | 70.6 ± 8.61 | 10 | 72.0 ± 8.19 |  |  |  |  |  |
|  |  |  |  |  |  |  | BMI(male)(kg/m^2^) | 117 | 28.13 ± 3.75 | 38 | 25.53 ± 4.06 |  |  |  |  |  |
|  |  |  |  |  |  |  | BMI(female)(kg/m^2^) | 30 | 26.99 ± 4.1 | 10 | 28.56 ± 5.46 |  |  |  |  |  |
|  |  |  |  |  |  |  | Smoking history(male) (package/year) | 117 | 38.83 ± 33.34 | 38 | 37.38 ± 32.39 |  |  |  |  |  |
|  |  |  |  |  |  |  | Smoking history(female) (package/year) | 30 | 18.63 ± 21.09 | 10 | 20 ± 15.52 |  |  |  |  |  |
|  |  |  |  |  |  |  | FEV_1_%(male) | 117 | 77.12 ± 18.53 | 38 | 63.68 ± 18.5 |  |  |  |  |  |
|  |  |  |  |  |  |  | FEV_1_%(female) | 30 | 88.13 ± 21.67 | 10 | 84.3 ± 33.09 |  |  |  |  |  |
|  |  |  |  |  |  |  | FVC%(male) | 117 | 72.36 ± 18.91 | 38 | 57.37 ± 15.77 |  |  |  |  |  |
|  |  |  |  |  |  |  | FVC%(female) | 30 | 85.9 ± 21.71 | 10 | 82.0 ±31.43 |  |  |  |  |  |
|  |  |  |  |  |  |  | FEV_1_/FVC%(male) | 117 | 83.63 ± 7.24 | 38 | 85.31 ± 8.76 |  |  |  |  |  |
|  |  |  |  |  |  |  | FEV_1_/FVC%(female) | 30 | 85.35 ± 7.18 | 10 | 85.5 ± 7.8 |  |  |  |  |  |
| 7 | Fujikawa T, et al[23] | 2022 | Japan | retrospective | IPF | 117 | Age(year) | 87 | 73.9 ± 7.8 | 30 | 76.6 ± 7.4 | 2.37 | 1.3 | 4.53 | median＞31 | lowest quartile of pectoralis muscle index |
|  |  |  |  |  |  |  | BMI(kg/m^2^) |  | 23.6 ± 3.3 |  | 20.7 ± 3.6 |  |  |  |  |  |
|  |  |  |  |  |  |  | Smoking history(package/year) |  | 39.8 ± 44.3 |  | 33.5 ± 37.8 |  |  |  |  |  |
|  |  |  |  |  |  |  | FEV_1_% |  | 73.9 ± 19.3 |  | 65.5 ± 16.6 |  |  |  |  |  |
|  |  |  |  |  |  |  | FVC% |  | 70.7 ± 19.5 |  | 63.8 ± 19.5 |  |  |  |  |  |
|  |  |  |  |  |  |  | DLCO% |  | 53.3 ± 20.8 |  | 38.5 ± 26.6 |  |  |  |  |  |
| 8 | Hanada M, et al[8] | 2022 | Japan | prospective | ILD | 78 | Age^*^(year) | 53 | 73.48 ± 11.43 | 25 | 71.4 ± 7.62 | NA | NA | NA | NA | AWGS 2019 |
|  |  |  |  |  |  |  | BMI^*^(kg/m^2^) |  | 24 ± 3.05 |  | 23.72 ± 6.29 |  |  |  |  |  |
|  |  |  |  |  |  |  | GAP index |  | 1.35 ± 0.76 |  | 1.64 ± 0.79 |  |  |  |  |  |
|  |  |  |  |  |  |  | FEV_1_%^*^ |  | 89.29 ± 18.29 |  | 87.14 ± 26.73 |  |  |  |  |  |
|  |  |  |  |  |  |  | FVC%^*^ |  | 85 ± 19.81 |  | 79.99 ± 23.58 |  |  |  |  |  |
|  |  |  |  |  |  |  | FEV_1_/FVC%^*^ |  | 78.35 ± 9.91 |  | 79.64 ± 11.79 |  |  |  |  |  |
|  |  |  |  |  |  |  | DLCO%^*^ |  | 61.77 ± 26.67 |  | 65.71 ± 29.87 |  |  |  |  |  |
|  |  |  |  |  |  |  | 6MWD(m) |  | 437.4 ± 98.3 |  | 403.8 ± 158.0 |  |  |  |  |  |
| 9 | Hagiwara H, et al[27] | 2025 | Japan | prospective | IPF | 306 | Age (years) | 229 | 72.0 ± 7.3 | 77 | 74.9 ± 6.3 | 1.61 | 1.11 | 2.50 | 60 | The cross-sectional area of the PM at T4 and ESM at T12 was manually marked and measured using Slicer 5.6.2 on 5-mm collimation CT scans obtained at registration. |
|  |  |  |  |  |  |  | Smoking history(package/year)^*^ |  | 40.0 ± 25.27 |  | 40.35 ± 29.46 |  |  |  |  |  |
|  |  |  |  |  |  |  | FVC % |  | 85.3 ± 19.4 |  | 76.1 ± 22.6 |  |  |  |  |  |
|  |  |  |  |  |  |  | FEV1 % |  | 85.3 ± 19.9 |  | 79.9 ± 21.7 |  |  |  |  |  |
|  |  |  |  |  |  |  | DLCO % |  | 65.4 ± 24.4 |  | 48.3 ± 29.8 |  |  |  |  |  |
| 10 | Cabrera-César E, et al[26] | 2025 | Spain | prospective | IPF | 84 | Age (years) | 67 | 70.21 ± 7.07 | 17 | 73.90 ± 7.19 | NA | NA | NA | NA | EWGSOP2 |
|  |  |  |  |  |  |  | BMI(kg/ m²) |  | 27.61 ± 3.43 |  | 27.61 ± 3.43 |  |  |  |  |  |
|  |  |  |  |  |  |  | ASMI |  | 7.31 ± 0.73 |  | 6.44 ± 0.46 |  |  |  |  |  |
|  |  |  |  |  |  |  | HGS(kg) |  | 36.56 ± 7.84 |  | 20.19 ± 4.39 |  |  |  |  |  |
|  |  |  |  |  |  |  | 6 MWD(m) |  | 417.05 ± 61.16 |  | 384.60 ± 78.77 |  |  |  |  |  |
|  |  |  |  |  |  |  | DLCO% |  | 48.23 ± 14.41 |  | 54.07 ± 24.46 |  |  |  |  |  |
| 11 | Fujita K, et.al[32] | 2024 | Japan | prospective | IPF | 64 | NA | | | | | 1.473 | 0.449 | 4.827 | 12 | AWGS 2019 |
| 12 | Ibarra-Fernández AA, et al[7] | 2025 | Mexico | prospective | ILD | 98 | NA | | | | | 2.3 | 1.3 | 4.06 | median = 50.4 | EWGSOP2 |
| 13 | Sridhar M, et.al[11] | 2024 | USA | prospective | IPF | 64 | NA | | | | | 2.1 | 0.8 | 5.3 | 6－12 | low FFMI and low hand grip strength |
| 14 | Okada A,et al[28] | 2026 | Japan | prospective | IPF | 63 | NA | | | | | 1.389 | 0.830 | 3.083 | 36 | AWGS2019 |

^*^: Obtained through algorithms; ^a^:Sarcopenia measured by the rectus femoris cross-sectional area; ^b^: Sarcopenia measured by the rectus femoris Y Axis; ^c^: Sarcopenia measured by the Timed Up & Go; DI-ILD: drug-induced interstitial lung disease

Supplementary Figure.1 Plot for the assessment of heterogeneity among the included studies of Age through leave-one-out elimination method

Supplementary Figure.2 Plot for the assessment of heterogeneity among the included studies of Smoking history through leave-one-out elimination method

Supplementary Figure.3 Plot for the assessment of heterogeneity among the included studies of GAP score through leave-one-out elimination method

Supplementary Figure.4 Plot for the assessment of heterogeneity among the included studies of FEV_1_% through leave-one-out elimination method

Supplementary Figure.5 Plot for the assessment of heterogeneity among the included studies of FVC% through leave-one-out elimination method

Supplementary Figure.6 Plot for the assessment of heterogeneity among the included studies of FEV_1_/FVC% through leave-one-out elimination method

Supplementary Figure.7 Plot for the assessment of heterogeneity among the included studies of DLCO% through leave-one-out elimination method

Supplementary Figure.8 Plot for the assessment of heterogeneity among the included studies of BMI through leave-one-out elimination method

Supplementary Figure.9 Plot for the assessment of heterogeneity among the included studies of HGS through leave-one-out elimination method

Supplementary Figure.10 Plot for the assessment of heterogeneity among the included studies of 6MWD through leave-one-out elimination method

Supplementary Figure.11 Plot for the assessment of heterogeneity among the included studies of ASMI through leave-one-out elimination method

Supplementary Figure.12 Plot for the assessment of heterogeneity among the included studies of Mortality through leave-one-out elimination method
